# Distinguishing between Clausius, Boltzmann and Pauling Entropies of Frozen Non-Equilibrium States

**DOI:** 10.3390/e21080799

**Published:** 2019-08-15

**Authors:** Rainer Feistel

**Affiliations:** Leibniz Institute for Baltic Sea Research IOW, 18119 Rostock, Germany; Rainer.Feistel@io-warnemuende.de

**Keywords:** empirical entropy, statistical entropy, residual entropy, Nernst theorem, Pauling entropy, metastable states, non-equilibrium, frozen states, symbolic information, Shannon entropy

## Abstract

In conventional textbook thermodynamics, entropy is a quantity that may be calculated by different methods, for example experimentally from heat capacities (following Clausius) or statistically from numbers of microscopic quantum states (following Boltzmann and Planck). It had turned out that these methods do not necessarily provide mutually consistent results, and for equilibrium systems their difference was explained by introducing a residual zero-point entropy (following Pauling), apparently violating the Nernst theorem. At finite temperatures, associated statistical entropies which count microstates that do not contribute to a body’s heat capacity, differ systematically from Clausius entropy, and are of particular relevance as measures for metastable, frozen-in non-equilibrium structures and for symbolic information processing (following Shannon). In this paper, it is suggested to consider Clausius, Boltzmann, Pauling and Shannon entropies as distinct, though related, physical quantities with different key properties, in order to avoid confusion by loosely speaking about just “entropy” while actually referring to different kinds of it. For instance, zero-point entropy exclusively belongs to Boltzmann rather than Clausius entropy, while the Nernst theorem holds rigorously for Clausius rather than Boltzmann entropy. The discussion of those terms is underpinned by a brief historical review of the emergence of corresponding fundamental thermodynamic concepts.

*Entropy is the most important quantity in the physics of heat.*

*Charles Kittel* [1]

*John von Neumann mentioned that ‘nobody knows what entropy really is’.*

*Jürn W. P. Schmelzer, Timur V. Tropin* [2]

## 1. Introduction

**Figure 1:**
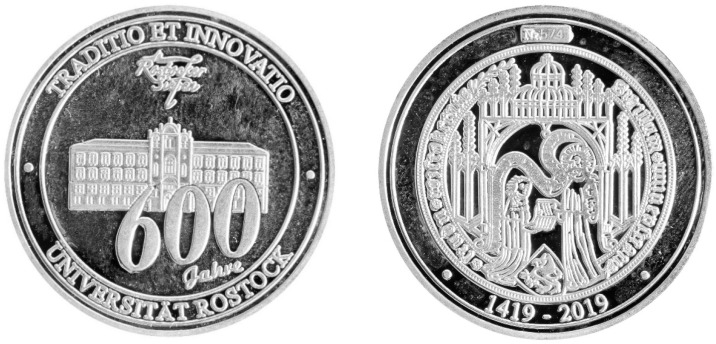
Silver medal issued at the 600th anniversary of the foundation of the Rostock University.

In 2019, the Rostock University is celebrating the 600th anniversary of its foundation, as the oldest such institution in the Baltic Sea region. On this occasion, a special silver medal has been issued recently (Figure 1). A century ago, on the occasion of the 500th jubilee, Einstein had visited the university when receiving the academic title of Dr. h.c. (Grambow [3]) which, by the way, was never formally rescinded later on, in contrast to most other German universities. Apparently, at that time Einstein [4] had been the first scientist who seriously considered the possible existence of a residual zero-point entropy of certain crystals at equilibrium (Gutzow and Schmelzer [5,6,7], see Appendix B).

It is clear that at given temperature and pressure, the mass of a metallic coin has a certain entropy, or let us better say, “bulk” entropy. To find a numerical value for this entropy, the coin may be heated up over a certain temperature range and the required energy may be measured. Then, a temperature integral over the recorded heat capacity results in the coin’s *Clausius entropy*, *S*_C_, as discussed in Section 2. Another method is by analysing the crystal lattice of the metal and estimating the number of quantum states the atoms may occupy. From this figure we obtain the coin’s *Boltzmann entropy*, *S*_B_, as discussed in Section 3. The two entropies turned out to be mutually consistent and may only differ, within the uncertainties of the methods applied, by a “trivial additive constant” (Planck [8]). Suitably adjusting this constant may lead to a vanishing zero-point entropy, commonly known as the 3rd law of thermodynamics, or the Nernst theorem, that had actually been formulated by Planck [9] in 1911 in going beyond the original hypothesis of Nernst [10] in 1906, see Appendix A. It was discovered later that, apparently violating the 3rd law, there may also exist a non-zero residual entropy at 0 K, termed the *zero-point entropy*, as introduced by Pauling [11] in 1935, see Appendix B.

However, the essential feature of a minted coin may not only be the material it is made of, rather, the shape of its surface and the symbolic information carried by this structure may be even more important. None of the methods mentioned above covers in an explicit way this fundamental aspect of certain thermodynamic systems, namely, of frozen, metastable non-equilibrium states. In this paper, therefore, the *Pauling entropy*, $S_{P}$, will be defined for non-equilibrium states in a more general way than being just the zero-point entropy, $S_{P}^{0}$, see Section 4. This definition refers to the fundamental non-equilibrium gap between the statistical entropy, which is super-additive with respect to the subsystems (Section 3), and the empirical entropy, which in contrast is additive (Section 2). Due to the 2nd law, this gap will asymptotically be filled by irreversible processes, but if the related Onsager fluxes are sufficiently slow, such as at frozen states, the gap may persist over a given observation time. For more details, an explicit, tutorial example for the calculation of this Pauling entropy of a metastable spatial configuration at a finite temperature is presented in Appendix C.

In his detailed review, Gujrati ([12], pp. 714, 716) also carefully distinguishes between two kinds of entropies, the “thermodynamic entropy” and the “statistical entropy”, explaining that “there is at present no consensus about their equivalence when the system is out of equilibrium”. A statistical entropy formulation is introduced in that article that is shown to be equivalent to the thermodynamic non-equilibrium entropy under relatively general conditions. This paper, in contrast, discusses the difference between the statistical micro-canonical Boltzmann entropy and the empirical Clausius entropy as a useful quantitative measure of frozen structures in non-equilibrium systems which are in a state of local equilibrium. These two entropies may differ already at equilibrium by the zero-point entropy, Appendix B, which cannot be measured empirically, i.e., without assuming a molecular microstructure. For non-equilibrium states, this difference is enhanced by the super-additivity of the Boltzmann entropy, giving rise to the generalized Pauling entropy.

In this paper, considerations are restricted to non-equilibrium systems that are in a state of *local equilibrium*. This means that the system’s volume may be divided into spatial cells that are sufficiently small (but still macroscopic) so that each cell can reasonably be assumed to be in internal equilibrium, while not necessarily being in mutual equilibria with adjacent cells. For non-equilibrium systems in local equilibrium, such as a solid exhibiting a temperature gradient, thermodynamic properties remain well-defined and measurable locally (Glansdorff and Prigogine [13], Falkenhagen and Ebeling [14], Subarew [15], Ebeling and Feistel [16], De Groot and Mazur [17], Feistel [18]). Restrictions to local equilibrium permit the easy use of temperature and entropy as thermodynamic properties. For other system, one cannot refer to textbook equilibrium definitions of those quantities, and novel definitions are indispensable, valid for those systems without local-equilibrium properties. As a simple counterexample to local equilibrium, an ideal-gas volume expanding freely into vacuum will lose its local equilibrium as its particles separate spatially by their velocities into spherical layers. Another important counterexample may be glasses if their local entropies—as far as those are well-defined at all—do not possess the properties of equilibrium entropies. Below a certain glass-transition temperature, typical glasses are frozen-in non-equilibrium systems (Gutzow and Schmelzer [5]), globally as well as locally. Therefore, care must be taken when thermal quantities and their mutual relations defined in irreversible local-equilibrium thermodynamics are borrowed for the description of other, frozen-in non-equilibrium systems.

In the limit of zero temperature, local-equilibrium systems described in this paper are generally assumed to be in equilibrium also globally. Otherwise, thermodynamically paradoxical situations may be encountered in which Clausius entropy apparently violates the 2nd law, see Section 2.

It is a fraction of the Pauling entropy that may reasonably be used for quantifying the information capacity of frozen states in the sense of the *Shannon entropy*, *S*_S_ (Shannon [19], Feistel [20]), see Section 5. In addition to the long list of available other publications on information entropy, this paper includes a discussion of appropriate relations between the different entropy definitions with respect to information. For this reason, those entropies will carry different subscripts here, in contrast to their otherwise conventional common symbol *S*.

As it was emphasized especially in the context of Synergetics, observers and modelers of physical systems are typically confronted with widely varying timescales of related processes (Hahn [21], Haken [22], Feistel and Ebeling [23]). Relaxation times of some modes (the “slaved” ones) may be much shorter than resolved by observation, others (the “control parameters”) may be too slow to watch them changing during the observation time, while those of most interest (the “order parameters”) range in between those extremes. This scenario applies as well to thermodynamic systems during their approach to equilibrium (Feistel [20], Gujrati [12]). Typically, the microscopic particle velocities adjust quickly to a Maxwell distribution, and the substance accordingly to a local equilibrium state. Irreversible processes such as Onsager fluxes of, say, thermal conduction or diffusion produce entropy and converge toward equilibrium (Glansdorff and Prigogine [13], De Groot and Mazur [17], Feistel and Ebeling [23]). Certain macroscopic structures, however, in particular of solids, see Figure 1, or spatially extended solutions like density-stratified oceans, remain virtually unchanged for extremely long periods of time, such as do mountain rocks or printed books, furniture or ceramics (Gutzow and Schmelzer [5,7]). In this paper, virtually stable states away from thermodynamic equilibrium which do not undergo any spontaneous processes (except insignificant fluctuations) within the given observation period are denoted as *metastable states*. Evidently, metastability in this subjective sense depends on the particular observers and their intentions. Such metastable systems are considered here as being trapped in *frozen non-equilibrium states*. Somewhat paradoxically, in this context, supercooled liquid water may also be regarded as “frozen”, contrary to its proper equilibrium state, namely ice (Handle et al. [24]). As well, chemical reactions may remain virtually locked in metastable states sometimes called “frozen equilibria” (Guggenheim, [25] (§1.48)). Note, however, that there exist alternative definitions for the terms ‘metastability’ and ‘frozen state’ that may qualitatively classify the physical details of different mechanisms hampering a quick relaxation to equilibrium (Gutzow and Schmelzer [7], Schmelzer and Tropin [26]).

**Figure 2:**
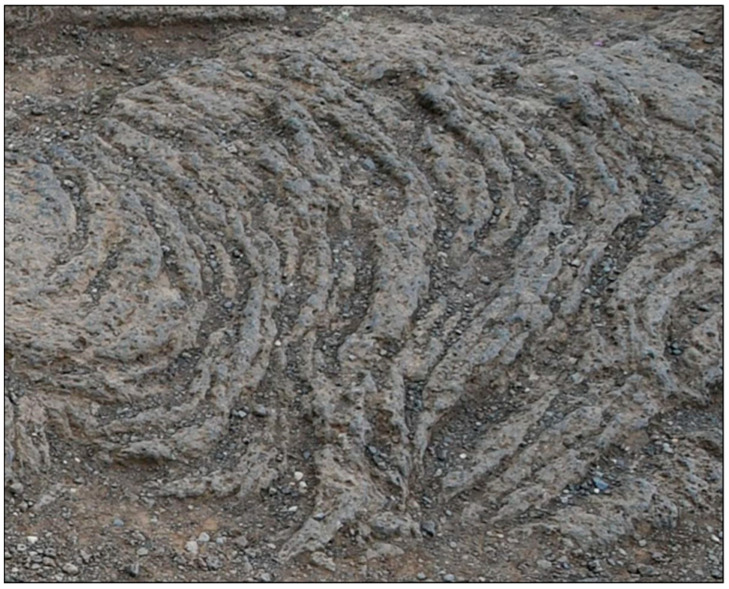
Frozen flow patterns of lava at Hraunfossar, Iceland. Photo taken in July 2019.

Proper equilibrium states are unique; at given energy and volume, they are the ultimate asymptotic target state for systems isolated from environmental influences, and do neither undergo spontaneous, natural changes nor do they preserve traces of the states they had taken in the past (Feistel and Ebeling [23]). There exist, however, energetically degenerate equilibrium states with certain degrees of freedom which are not uniquely dictated by the entropy maximum or some energy minimum. For example, of a liquid-vapour equilibrium in the absence of gravity, the two phases may occupy different configurations in space, depending on the system’s past. Similarly, frozen non-equilibrium states are not necessarily unique and may constitute a petrified record of the time history of their formation processes (Figure 2). Such structures are information carriers, and the question arises to what extent the entropy of their states may serve as a measure of their information content.

There are two qualitatively different kinds of information potentially stored or transferred by metastable systems, namely, structural and symbolic information (Feistel and Ebeling [23,27], Feistel [28], Burgin and Feistel [29]). An example for structural information are the layers of different rocks in the Earth’s crust that Darwin [30] (p. 321) had regarded “as a history of the world imperfectly kept and written in a changing dialect”. On the other hand, symbolic information is coded arbitrarily in physical structures (Pattee [31]) such as the letters or numbers that shape the surface of metallic coins (Figure 1) or tombstones (Figure 4). Both kinds of information ultimately disappear by “weathering” from a frozen non-equilibrium system in the course of its relaxation to equilibrium.

## 2. Clausius Entropy

In 1854, when studying cyclic thermal processes, Rudolf Clausius discovered that the integral over the exchanged heat, d*Q*, of a given body must always vanish for reversible processes, independently of the way the cycle is conducted ([32], p. 93):

(1) $$\oint \frac{dQ}{T}=0$$

He concluded that there exists a new state quantity, *S*_C_, which he termed *entropy*, defined by its differential d*S*_C_ = d*Q*/*T*. The subscript “C” at *S* has been attached here indicating “Clausius”. This quantity can be calculated at given volume or pressure, respectively, from the related measurable isochoric or isobaric heat capacities, *C_v_* or *C_p_*, respectively, by the following expressions (Clausius [32], pp. 214–215):

(2) $$S_{C}\left(T,V\right)=\int_{T_{\mathrm{ref}}}^{T}\frac{C_{v}\left(T^{\prime },V\right)}{T^{\prime }}dT^{\prime }\;\mathrm{or}\;S_{C}\left(T,p\right)=\int_{T_{\mathrm{ref}}}^{T}\frac{C_{p}\left(T^{\prime },p\right)}{T^{\prime }}dT^{\prime }.$$

Here, *T*_ref_ is an unspecified reference temperature that gives rise to an arbitrary integration constant in the definition of Clausius entropy. It was assumed in 1906 by Planck ([8], p. 137) that this additive constant “has no physical meaning and may be omitted at will”. Also in 1906, however, Walther Nernst published his theorem, expressing in the words of Planck ([9], p. 268) in 1911 that “at the zero point of the absolute temperature, the entropy of any chemically homogeneous solid or liquid body possesses a certain value which is independent of its phase and its particular chemical modification”, see Appendix A. Planck ([9], p. 270) concluded that without loss of generality, entropy can be defined by:

(3) $$S_{C}\left(T,p\right)\equiv \int_{0}^{T}\frac{C_{p}\left(T^{\prime },p\right)}{T^{\prime }}dT^{\prime }$$

Of the two possible choices, Equation (2), Planck preferred pressure as the second variable for its ease of measurement and its equality across phase boundaries. Equivalently, however, one may exploit the alternative isochoric option instead:

(4) $$S_{C}\left(T,V\right)=\int_{0}^{T}\frac{C_{v}\left(T^{\prime },V\right)}{T^{\prime }}dT^{\prime }$$

for its comparability with the independent variables of the canonical statistical ensemble.

Equation (4) serves as the definition of Clausius entropy in this paper. Note that definitions formally deviating from Equation (4) may be considered elsewhere for certain reasons, such as specifying *T*_ref_ in Equation (2) by the triple point of water in order to reduce the uncertainty of empirical equations (Feistel [18]), or by the melting temperature of metastable, glass-like solids. Such arbitrary definitions, if deviating from Equation (4) by merely a numerical constant, do neither affect any measurable thermodynamic properties nor the physical description of natural processes.

For the existence of the integrals (3), (4) it is necessary that both *C_p_* and *C_v_* vanish at *T* = 0. This is evidently not the case, however, for ideal gases with constant heat capacities, for which entropy diverges logarithmically at the zero point (Fermi [33] (p. 147)). Gases may be cooled down to very low temperatures without condensation as long as the lowered pressure does not exceed the saturation or sublimation pressure. For water vapour, for example, the sublimation pressure at 23 K amounts to 10^−100^ Pa, and the resulting (theoretical) ideal-gas density is as low as a single molecule in the entire universe (Feistel and Wagner [34]). It is clear that such a dilute gas cannot exist in any lab, and that icy comets far from the sun cannot evaporate, even in the emptiest cosmic void.

**Figure 3:**
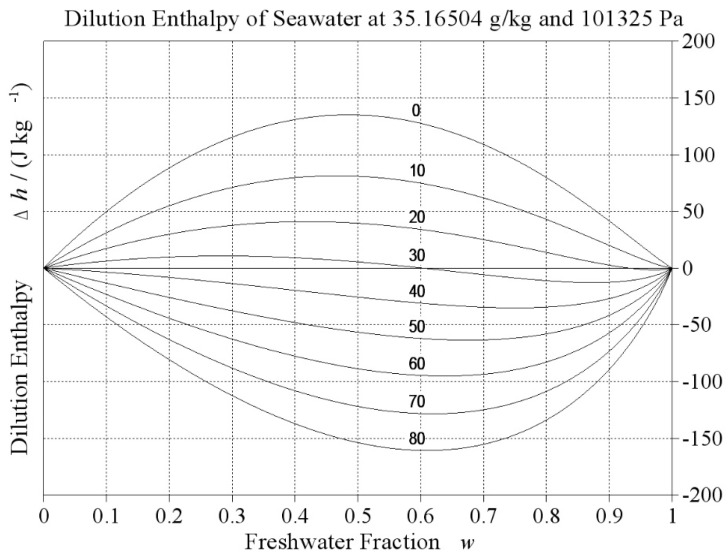
Specific dilution enthalpy of ocean surface water when admixed with freshwater (rain, melting ice, river discharge) for different freshwater fractions, *w*, and temperatures in °C as indicated by the curves (Feistel [35]). Diagram computed from the TEOS-10 Gibbs function of seawater (Feistel [18,36]).

For the entropy of metastable states it must be emphasized that Equation (1) is formulated for reversible processes, and that the entropy definitions (3) and (4) are valid only for equilibrium states and frozen metastable states. This restriction becomes evident if Equation (4) is written in differential form:

(5) $$dS_{C}\left(T,V\right)=C_{v}\;dT/T$$

which implies that under reversible conditions, Clausius entropy can only grow along with rising temperature. Experiments show, however, that there exist systems that cool down spontaneously while they relax to equilibrium, producing entropy. Especially electrolyte solutions (Falkenhagen and Ebeling [14], chapter 8.6) may possess negative dilution enthalpies, see Figure 3, so that the irreversible mixing of two solutions with different concentrations may result in a mixture with lower temperature but higher entropy. As another example, when liquid water is brought in contact with sub-saturated vapour, some water will evaporate and the absorbed latent heat will result in a colder final state, such as when sweating in dry, hot air.

Heat capacity is primarily quantifying a relation between temperature and energy, rather than entropy. Under the weak assumptions that a given system is confined to a certain fixed volume *V* and possesses a well-defined temperature *T*, the system’s heat capacity can be determined empirically from the power supplied to an electrical heater, d*U*, and the resulting temperature rise, d*T*:

(6) $$dU=C_{V}\;dT.$$

No special knowledge of the system’s internal details is needed for conducting such a measurement. In turn, energy conservation relates *C_V_* to the entropy via the Gibbs fundamental equation:

(7) $$dU=T\;d_{e}S_{C}$$

In contrast to Equation (5), the total change of entropy related to an irreversible transition, however, includes an additional term, *P*, of entropy production (Guggenheim [25], equation 1.17.1):

(8) $$dS_{C}=\;d_{e}S_{C}+d_{i}S_{C}=\frac{dU}{T}+Pdt$$

For the general, irreversible case, therefore, the Planck definition (4) of the Clausius entropy may be generalised by including additional terms that describe entropy production (De Groot and Mazur [17]):

(9) $$P=\sum X_{k}J_{k}={\left(\frac{\partial S_{C}}{\partial \xi }\right)}_{U,V}\frac{d\xi }{dt}.$$

Here, ξ is a set of internal parameters that describe the system’s non-equilibrium structures and approach ξ=0 at equilibrium, along with disappearance of the related Onsager forces *X_k_* and fluxes *J_k_*. For equilibrium states, *P* vanishes by definition, and for frozen metastable states, the observation period t is short enough for reasonably neglecting the entropy production term, so that Equation (8) reduces to Equation (4) in these special cases.

The [Clausius] entropy of a system of bodies in different states is the sum of the entropies of each of the bodies (Maxwell [37] (p. 163)). Similarly, Planck ([9], p. 100) also defined the total (Clausius) entropy of a composed system as additive: “Endlich bezeichnen wir die Summe der Entropien mehrerer Körper kurz als die Entropie des Systems aller Körper.” Following Planck, this statement holds for any decomposition as long as the parts possess well-defined homogeneous, while possibly mutually different, temperatures and densities, regardless of their barycentric velocities. Such a local-equilibrium approach is consistent with modern conventional irreversible thermodynamics (Glansdorff and Prigogine [13], Falkenhagen and Ebeling [14], De Groot and Mazur [17], Feistel and Ebeling [23]). Also, entropy production of non-equilibrium, local-equilibrium states is additive (De Groot and Mazur [17], equation III.8).

Imagine a set of subsystems at *T* = 0, each separately being in internal equilibrium and possessing zero Clausius entropy according Planck’s definition (4) of a universal absolute entropy at the zero point. The additivity of Clausius entropy with respect to the body’s subsystems, as expressed by Maxwell, Planck and also by the common local-equilibrium thermodynamics, then results in a total entropy of *S*_C_ = 0. This value remains unchanged regardless of whether or not those subsystems are in mutual equilibria. Consequently, local-equilibrium systems at *T* = 0 possess the same total Clausius entropy for global equilibrium and non-equilibrium states, a conclusion which apparently violates the 2nd law. To circumvent such paradoxical properties, at *T* = 0 the Clausius entropy definition of this paper may not be applied to systems that are in non-equilibrium configurations.

The fundamental additivity of Clausius entropy may be expressed by a formal definition:

*For any two parts of a thermodynamic system with additive volumes*, *V*(1 + 2) = *V*(1) + *V*(2), *and additive internal energies*, *U*(1 + 2) = *U*(1) + *U*(2), *the Clausius entropy is additive*:

(10) $$S_{C}\left(1+2\right)=S_{C}\left(1\right)+S_{C}\left(2\right)$$

To prevent possible misunderstandings, it cannot be concluded from Equation (4) that additivity of *S*_C_ might imply additivity of *C*_V_. As a counterexample, while the entropy of sea ice or of wet air is additive, the heat capacities of those composites include additional contributions of latent heat due to compositional changes with temperature (Feistel et al. [38]). The total heat capacity, in addition to the sum of the heat capacities of the system’s constituents, may also include latent contributions from phase transitions and mass transfers between those parts, even during reversible processes.

For thermodynamic stability, heat capacities are nonnegative. For the 2nd law, entropy production is nonnegative. So, Clausius entropy, Equation (9), is nonnegative:

(11) $$S_{C}\geq 0$$

For the 2nd law, Clausius entropy of a closed system cannot decrease during relaxation to equilibrium:

(12) $$\frac{dS_{C}}{dt}=P\geq 0$$

Clausius entropy may also be termed “thermal entropy”, or simply “entropy” as done by its mental father himself, or “thermodynamic entropy” as done by Simon [39], “caloric entropy” (Ebeling [40]), or “Clausius-Nernst-Planck entropy” for appreciating the definition (3).

## 3. Boltzmann Entropy

**Figure 4:**
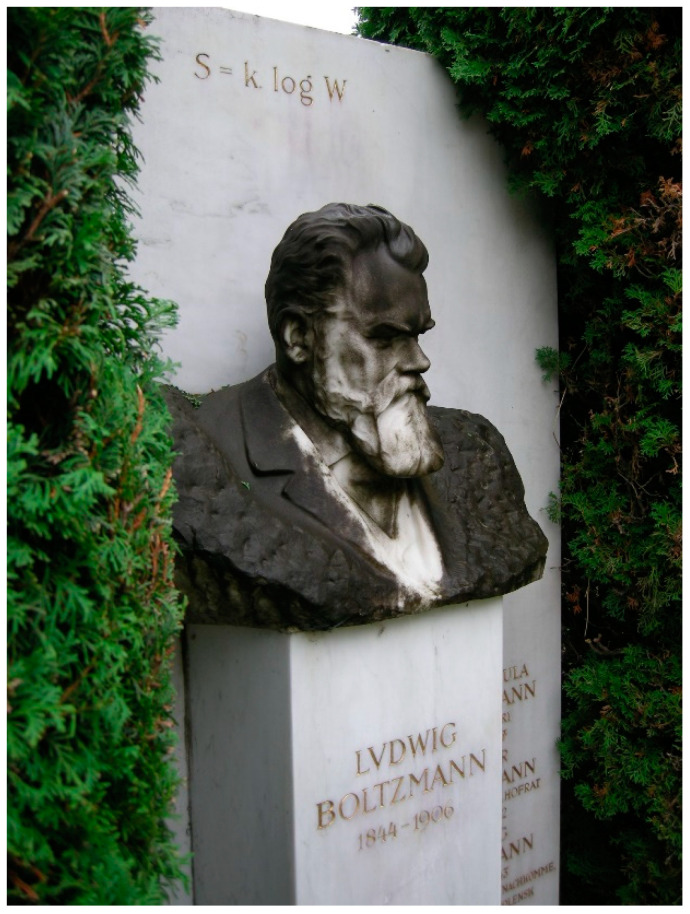
Boltzmann’s grave at Vienna’s central cemetery. Photo taken in October 2010.

Occasionally, just by concentrated contemplation, exceptional theoretical physicists have been able to establish surprisingly close but completely unforeseen mathematical links between previously unrelated quantities, such as Einstein with his famous equation *E* = *mc*^2^, or Planck’s invention of quantum mechanics with *E* = *hν*. At Boltzmann’s grave in Vienna, a similar, as fundamental as simple relation between (Boltzmann) entropy, *S*_B_, and number of microstates (also dubbed “thermodynamic probability” such as done by Planck in the quotation below), *W*, is encarved, see Figure 4:

(13) $$S_{B}\equiv k_{B}\ln W$$

This equation is the fundamental relation of the micro-canonical statistical ensemble that permits the calculation of entropy as a thermodynamic potential function in terms of the natural variables energy and volume from the number of quantum states possible at those given values. Actually, this equation was derived by Planck [8], including the Boltzmann constant, *k*_B_, with appreciation politely given to Boltzmann [41]. Later, Planck ([42], p. 119) explained the relation between his and Boltzmann’s works, which may be quoted here literally at length for elucidating the important question of the very authorship of the famous Equation (13): “Der logarithmische Zusammenhang zwischen Entropie und Wahrscheinlichkeit ist von L. Boltzmann aufgedeckt worden, in seiner kinetischen Gastheorie (Boltzmann [41], §6). Doch unterscheidet sich unsere Gleichung [8] ihrer Bedeutung nach in zwei wesentlichen Punkten von der Boltzmannschen. Erstens fehlt bei Boltzmann der Faktor [*k*_B_], was damit zusammenhängt, daß Boltzmann nicht mit den wirklichen Molekülen, sondern immer nur mit gr-Molekülen rechnete. Zweitens aber, was noch viel tiefgehendender ist, bleibt bei Boltzmann, wie überhaupt in der gesamten klassischen Thermodynamik, die Entropie [*S*_B_] hinsichtlich einer additiven Konstante unbestimmt und dementsprechend bleibt in dem Werte der Wahrscheinlichkeit *W* ein Proportionalitätsfaktor unbestimmt. Im Gegensatz dazu schreiben wir der Entropie [*S*_B_] eine ganz bestimmte absolute Größe zu. Das ist ein Schritt von prinzipieller Tragweite Er führt mit Notwendigkeit zur Quantenhypothese und dadurch einerseits zu einem bestimmten Energieverteilungsgesetz der schwarzen Strahlung, andererseits zum Nernstschen Wärmetheorem.“ Here, Planck had put particular emphasis to the fact that the formerly arbitrary absolute value of the empirical entropy becomes well-defined statistically, as a consequence of his Equation (13).

Chapter 6 of Boltzmann [41], as referred to by Planck [8] in this context, presents on its p. 38 the famous *H* theorem, expressed in terms of the single-particle distribution function, similar to Boltzmann’s [43] (equation 65) ideal-gas entropy formula. However, this preliminary “Boltzmann entropy” is valid only for ideal gases rather than for the general case (Subarew [15], p. 26). Therefore, Einstein [4] (p. 826) characterized Equation (13) as “das Boltzmannsche Prinzip in der Boltzmann-Planckschen Fassung“. Concluding, it was Planck who first published Equation (13) in its common very general form, although inspired by Boltzmann. In most modern textbooks, Equation (13) is taken for granted: “Die Entropie ist der Logarithmus der Anzahl der Zustände, die dem System möglich sind“ (Kittel [1], p. 61). For frozen non-equilibrium states, Boltzmann entropy is more relevant than Clausius entropy in the sense that statistical thermodynamics is richer than empirical thermodynamics by including also molecular information. “The entropy that such systems exhibit has, of course, a statistical meaning, but not a thermodynamic one” (Simon [39], p. 1094).

In a non-equilibrium situation of a body with given total values for energy *U*, volume *V* and particle number *N*, its initial entropy will be lower than at equilibrium, according to the 2nd law. However, those conditions given, the number *W*(*U*, *V*, *N*) of quantum states is uniquely fixed, and so the body’s Boltzmann entropy (13) will take its equilibrium value from the very beginning and is evidently incapable of increasing any further. What is changing, however, during irreversible relaxation is the occupation density of the available states, but this density does not enter the Boltzmann entropy formula (13). When considering two systems, both individually at equilibrium but not mutually, it is clear that entropy will be produced as soon as the two system come into contact. That is, new quantum states in addition to those existing initially will emerge immediately just by permitting interaction between the parts of the composite system, while the occupation of those initially empty microstates happens only gradually and delayed by irreversibly spreading into the newly opened pristine void. This means that Boltzmann entropy is super-additive for adhering the 2nd law. A rigorous proof of this super-additivity was given by Kittel ([1], p. 85).

If a system 1 with *W*_1_ microstates gets in contact with a system 2 with *W*_2_ microstates, the combined system possesses *W* = *W*_1_ *W*_2_ *W*_12_ microstates, where the newly emerging microstates, *W*_12_ ≥ 1, result from the additionally possible exchange of particles and energy between the systems 1 and 2, and give rise to the super-additive excess entropy, $S_{B}\left(1+2\right)-S_{B}\left(1\right)-S_{B}\left(2\right)=k_{B}\ln W_{12}$. Initially, when the two parts get in contact, those new microstates are not occupied and do neither contribute to the heat capacity nor, in turn, to the Clausius entropy of the combined system; they need irreversible exchange fluxes between parts 1 and 2 in order to gradually become visible experimentally as entropy production and increase of *S*_C_. In general, for local-equilibrium systems without zero-point entropy, the local Boltzmann entropies equal the local Clausius entropies. However, for such non-equilibrium states, the total Boltzmann entropy exceeds the particular sum of those local values, while the total Clausius entropy equals that sum.

The fundamental super-additivity of Boltzmann entropy may be expressed by a formal definition:

*For any two parts of a thermodynamic system with additive volumes*, *V*(1 + 2) = *V*(1) + *V*(2), *and additive internal energies*, *U*(1 + 2) = *U*(1) + *U*(2), *the Boltzmann entropy is super-additive*:

(14) $$S_{B}\left(1+2\right)\geq S_{B}\left(1\right)+S_{B}\left(2\right)$$

According to the Jensen [44] inequality, this implies that Boltzmann entropy is a convex function of the extensive state variables (Alberti and Uhlmann [45], equation 12, Lieb and Yngvason [46], equation 2.21).

By its definition, Boltzmann entropy is nonnegative, and Clausius entropy cannot exceed it:

(15) $$S_{B}\geq S_{C}\geq 0$$

Boltzmann entropy of a closed system does not increase during relaxation to equilibrium:

(16) $$\frac{dS_{B}}{dt}=0$$

From the perspective of this paper, there is another qualitative difference between the statistical entropy definition of Planck [8], Equation (13), and the preliminary Boltzmann [41] entropy. Planck’s definition, Equation (13), which is termed the Boltzmann entropy *S*_B_ here, counts the number of microstates regardless of their occupation dynamics and is, consequently, unrelated to the 2nd Law. Boltzmann’s [41] preliminary entropy, however, relies on the dynamics of microstate occupation, and obeys the 2nd law in the form of the famous H-theorem. The latter entropy is, accordingly, a statistical expression for the Clausius entropy, *S*_C_.

Boltzmann entropy may also be termed “statistical entropy”, as done by Simon [39], or “Boltzmann-Planck entropy” in the sense of Einstein [4] and appreciating Planck’s formulation (13).

## 4. Pauling Entropy

There are good physical reasons for the vanishing heat capacities of condensed bodies at *T* = 0, and consequently for the 3rd law to hold, *S*_C_ = 0. On the contrary, as already suspected by Einstein [4], rigorous physical reasons are lacking for an inevitable existence of a unique single quantum state, *W* = 1, at the zero point, and accordingly, for *S*_B_ = 0. Beginning with Boltzmann, Planck and Einstein, the traditional physical understanding is that entropy is one and the same physical quantity that may be calculated with different methods and formulas, such as Equations (3) and (13). Accordingly, it is often argued that the Nernst theorem is not applicable to frozen non-equilibrium systems which possess quantum states that count to the number *W* but do not contribute to the body’s heat capacity (Landau and Lifschitz [47], §61). “Der Satz von Nernst ist nicht … [anwendbar auf] ‘eingefrorene’ metastabile Zustände” (Subarew [15], p. 107)). Similarly, Gutzow and Schmelzer ([7], p. 63) conclude that “the Third law of thermodynamics and some of its consequences, as they are known for equilibrium systems, fail for the vitreous state“. This traditional view classifies systems with residual entropies as somewhat exceptional or irregular with respect to the Nernst theorem, such as glasses or other amorphous or metastable states.

In contrast to this common view, it is suggested here that Clausius und Boltzmann entropies may be considered as two different physical quantities that may coincide under certain circumstances but do not always need to. Regarded as the 3rd law of thermodynamics, the Nernst theorem naturally applies to *S*_C_ of local-equilibrium systems, but not necessarily to *S*_B_, as intentionally contrasted here to the zero-point entropy which may exist only for *S*_B_ but never for *S*_C_, according to the definition (4).

The difference between *S*_B_ and *S*_C_:

(17) $$S_{P}\equiv S_{B}-S_{C}\equiv k_{B}\ln\frac{W}{\Omega }$$

is generalized here to be valid for arbitrary temperatures, this way defining the *Pauling entropy*, originally defined only at the zero point, $S_{P}\left(T=0\right)\equiv S_{P}^{0}$, see Appendix B. Here, Ω denotes the number of quantum states responsible for the body’s measurable heat capacity, as defined by the Clausius entropy, $S_{C}=k_{B}\ln\Omega$. Conversely to Ω, the property *S*_P_ is a measure of the fraction of quantum states that *do not* contribute to its heat capacity. Irreversible processes convert Pauling entropy into Clausius entropy at constant Boltzmann entropy.

At equilibrium, the relation between heat capacity *C_v_* and microscopic quantum energy states *E_n_* is given by the canonical statistical Equation (Schrödinger [48], equation 5.10, Kittel [1], equation 6.63), Subarew [15], equation 13.8), Klimontovich [49], equation 6.4.4):

(18) $$C_{v}={\left(\frac{\partial }{\partial T}\right)}_{V}\langle E\rangle =\frac{1}{k_{B}T^{2}}\langle {\left(E-\langle E\rangle \right)}^{2}\rangle .$$

Here, the average <…> is weighted by Boltzmann factors of the canonical ensemble:

(19) $$\langle E\rangle =\frac{\sum_{n}E_{n}\exp\left(-\frac{E_{n}}{k_{B}T}\right)}{\sum_{n}\exp\left(-\frac{E_{n}}{k_{B}T}\right)}=U\left(T,V\right)$$

The energy levels *E_n_* depend on the volume *V* but not on the temperature. Quantum states that contribute to the Boltzmann entropy but do not contribute to the heat capacity, especially of local-equilibrium configurations, possess energy levels that may be removed from the corresponding sums (19) without affecting the total sum of local dispersions (18) of the energy distributions. It is expected that among those states are, in particular, terms with sufficiently high, thermally inaccessible “frozen” levels, $E_{n}\gg k_{B}T$.

If *S*_B_ equals the maximum Clausius entropy at equilibrium, i.e., if $S_{P}^{0}=0$, while the actual value of *S*_C_ describes that of a certain non-equilibrium state, Pauling entropy *S*_P_ equals the “entropy lowering” introduced by Klimontovich [49], describing the distance from equilibrium at the same energy and volume (Feistel and Ebeling [23]):

(20) $$S_{P}\left(U,V,t\right)=S^{\mathrm{eq}}\left(U,V\right)-S\left(U,V,t\right)=S_{B}\left(U,V\right)-S_{C}\left(U,V,t\right)$$

A similar term for this Pauling entropy is “negentropy” (Brillouin [50], Ebeling and Feistel [51]). In the general case with arbitrary $S_{P}^{0}$, integration of Equation (8) with d*U* = 0 results in a formula for estimating the Pauling entropy (see Appendix C):

(21) $$S_{P}\left(U,V,t\right)=S_{C}\left(U,V,\infty \right)+S_{P}^{0}-S_{C}\left(U,V,t\right)=\int_{t}^{\infty }Pdt+S_{P}^{0}$$

By the definition (17), Pauling entropy is suggested to advance conceptionally from some previous “exceptional statistical correction term” at the zero point, $S_{P}^{0}$, to a regular thermodynamic measure for frozen and other non-equilibrium states at finite temperatures, Equation (21). It is a measure of structure, organization and information contained in a non-equilibrium system (Klimontovich [49,52]) and expresses the difference between the “valoric” and “caloric” interpretations of entropy (Schöpf [53], Ebeling [40], Feistel and Ebeling [23]). An explicit tutorial example for this quantity, the amount of “potential” entropy that is possibly producible by future dissipation, Equation (21), is discussed in Appendix C.

Consistently with Equation (20) and the Gibbs [54] fundamental equation, the definition (17) is understood to be taken at fixed given values of *U*, *V*, ***N***:

(22) $$S_{P}\left(U,V,N,\xi \right)=S_{B}\left(U,V,N\right)-S_{C}\left(U,V,N,\xi \right)\equiv k_{B}\ln Q$$

Here, ***ξ*** is a set of macroscopic properties that characterise the non-equilibrium state such that ***ξ*** = 0 holds at equilibrium (Gutzow and Schmelzer [7], Gujrati [55]), see Equation (9), and ***N*** are suitably defined particle numbers of the constituents. The quantity Q(U,V,N,ξ) is a measure for the number of configurations possible at fixed (U,V,N,ξ) that do not affect the system’s heat capacity, *C*_V_. For example, in the case of a coin as shown in Figure 1, one may think of the number of spatial deformations and surface shapes that the same amount of metal may undergo. Typically, *Q* may be a relatively small number, that is, *Q* << *W*.

The physical interpretation of the quantity Ω = *W*/*Q* is subject to ongoing scientific discussion. For non-equilibrium states, in addition to energy and volume, Ω depends on further macroscopic state variables, ***ξ***. Onsager [56] used this dependence to demonstrate the symmetry of transport coefficients in linear irreversible thermodynamics (Landau and Lifschitz [57], §58). ”Ein makroskopischer Zustand umfaßt immer eine große Anzahl mikroskopischer Zustände, die [der thermodynamische Beobachter] zu einem Mittelwert zusammenfaßt” (Planck [42], p. 121). Accordingly, the quantity Ω(t) may be understood as an effective number of microstates that the system had visited within a reasonable observation period Δ*t* at the time *t*, or as a currently occupied part of the Boltzmann energy shell (Ebeling [40], Ebeling and Feistel [58], Feistel and Ebeling [23,27], Feistel [20]). Note that, according to Liouville’s theorem, the instantaneous phase volume, Ω_inst_, occupied by each successively taken single microstate is time-invariant even during irreversible processes (Gibbs [59] (equation 22), Planck [42] (§129), Subarew [15], §2.1, Klimontovich [49], p. 30). Therefore, the growing value of Ω(*t*), Equation (12), cannot be associated with either of the two quantities, *W* or Ω_inst_, rather, it is likely related to an average microstate number visited during the observation period, controlled by the magnitude of fast thermal and quantum fluctuations. However, physical arguments may be raised against a simple mechanical interpretation of Ω(*t*) being a number of visited microstates, or being a time average over visiting frequencies (Ishioka and Fuchikami [60], Goldstein [61]). In fact, microscopically, thermodynamic systems are quantum systems (Schrödinger [48]), and the question of whether or not a system is “really” in a certain microstate at some sharp instance of time may not make definite physical sense unless that state has actually been measured and its wave function has collapsed. While the existence and definition of Ω(*t*) is not in question here, obstreperous problems arise with the attempt of painting a simple, mechanical, common-sense picture of it. Bearing this precaution in mind, one may think of Ω(*t*) for simplicity as some average number of occupied microstates (Ufflink [62]).

From the definitions given for *S*_P_, *S*_B_ and *S*_C_, super-additivity auf Pauling entropy can be concluded:

*For any two parts of a thermodynamic system with additive volumes*, *V*(1 + 2) = *V*(1) + *V*(2), *and additive internal energies*, *U*(1 + 2) = *U*(1) + *U*(2), *the Pauling entropy is super-additive*:

(23) $$S_{P}\left(1+2\right)\geq S_{P}\left(1\right)+S_{P}\left(2\right)$$

Pauling entropy is nonnegative and cannot exceed Boltzmann entropy:

(24) $$S_{B}\geq S_{P}\geq 0$$

There is no general inequality valid between Pauling and Clausius entropies.

Expressing the devaluation of energy by dissipation, Pauling entropy of a closed system cannot increase during relaxation to equilibrium:

(25) $$\frac{dS_{P}}{dt}=-P\leq 0$$

The definition *S*_B_ = *S*_C_ + *S*_P_ suggests, in analogy to mechanical energy, a simple interpretation of Boltzmann entropy as a “total entropy”, Clausius entropy as a “kinetic entropy” and Pauling entropy as a “potential entropy”. Then, the relaxation to equilibrium can be read as a - possibly incomplete - transformation of potential entropy into kinetic entropy.

Assumingly, Pauling entropy as defined here (Feistel [20]) is closely related to the “configurational entropy” discussed recently in conjunction with various additional entropy measures relevant for glassy states (Berthier et al. [63]). A detailed analysis of different approaches to the determination of the configurational entropy in glass transitions is given by Schmelzer and Tropin [2]. Pauling entropy may also be termed “residual entropy”, “valoric entropy” (Ebeling [40]), “entropy lowering” or “Klimontovich entropy”.

## 5. Shannon Entropy

“In 1948, Shannon …. called his measure, as allegedly suggested by von Neumann: ‘entropy’. This proved to be a grievous mistake which had caused great confusion in both information theory and thermodynamics” (Ben-Naim [64], p. 1). This accusation sounds even more severe when taking into account that even “John von Neumann mentioned that ‘nobody knows what entropy really is’” (Schmelzer and Tropin [2], p. 1, referring to Petz [65]). In fact, the relation between Shannon’s information entropy and thermodynamic entropy is still subject to controversial opinions and discussions in the scientific literature (Ebeling and Feistel [51]).

**Figure 5:**
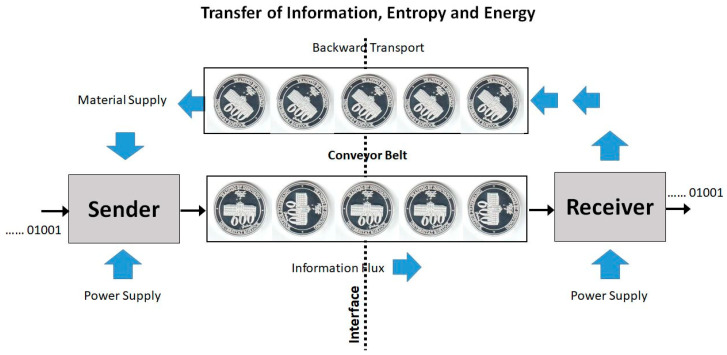
Model for information transfer across an interface by a sequence of symbols in the sense of Shannon [19] for comparison with thermodynamic properties of the message and of the information carrier (Feistel [20]).

Imagine you give your memory stick to a colleague who copies a file onto it and returns it to you. How much energy or entropy must inevitably be exchanged together with the desired information? A fictitious, conceptional model for such a symbolic information transfer, Figure 5, may illustrate the relation between the Shannon [19] entropy, *S*_S_, and other entropies. The information source is turning a coin in either a 0° position (indicating 0) or a 90° position (indicating 1). The coin is placed on a conveyor belt, along with additional copies of the coin, each turned appropriately to form the message, and transported to a receiver. There, the information bit carried by the coin is read and erased by turning the coin in a neutral 45° position, and conveying the coin back to the sender, replenishing the raw material stock. The coin’s energy is degenerate with respect to different turning angles; such neutral or Goldstone modes (Obukhov [66], Pruessner [67]) are characteristic for symbolic information carriers (Feistel and Ebeling [23], Feistel [20]). There is no serious doubt that such a technical machine is capable of transferring messages from the transmitter to the receiver. Regardless of that, the model exhibits some rigorous thermodynamic properties:

(i) Across the interface, there is no net energy flux sustained by the conveyor belt. Each coin is carried back and forth with the same internal energy and the same potential energy in the gravity field.
(ii) Across the interface, there is no net Clausius entropy flux sustained by the conveyor belt. Each coin is carried back and forth with the same Clausius entropy, which is additive to form the Clausius entropy of the message.
(iii) Across the interface, there is no net single-coin Boltzmann entropy flux sustained by the conveyor belt. Each coin separately is carried back and forth with the same Boltzmann entropy, despite its actual orientation. The super-additive Boltzmann entropy of the sequence of coins applies to both transport directions independently of the actually transferred message.
(iv) Across the interface, there is no net single-coin Pauling entropy flux sustained by the conveyor belt. Each coin separately is carried back and forth with the same Pauling entropy, despite its actual orientation. Also the Pauling entropy of the set of coins does not depend on their actual individual turning angles.

In addition to the other entropies, also the Shannon entropy (in the sense of information capacity) is the same for the messages communicated forward and backward; the only difference is that the one message is meaningful and read by the receiver while the latter one is meaningless and ignored upon return to the sender. Evidently, this difference is of technical rather than thermodynamic nature. While information exchange between two systems necessarily requires a physical carrier facilitating at least two alternative symbols, the net flux of information is not necessarily accompanied by net fluxes of entropy or energy.

The Pauling entropy of the coin is a measure for the number of possible spatial configurations of the coin’s material at constant total energy and volume, and possibly additional macroscopic non-equilibrium properties, see Section 3 and Section 4. Rotating the anisotropically formed coin by a certain angle such as shown in Figure 5 results in a “modified” coin which also belongs to the set of alternative configurations that count to the Pauling entropy of the metastable, frozen-in solid. Correspondingly, different turning angles displayed by a set of coins belong to the Pauling entropy of that set, that is, to the total Pauling entropy of the message. Of those angles and configurations, by sender and receiver only a certain subset is jointly understood as the set of supported symbols, the “alphabet”, for information transfer. The number *M* of differently defined, conventional symbols is therefore bound from above by the number *Q* that specifies the Pauling entropy. The Shannon entropy defined in thermodynamic units rather than in bits:

(26) $$S_{S}\equiv k_{B}\ln M$$

cannot exceed the Pauling entropy of thermodynamic structures used as symbols, that is:

(27) $$S_{B}\geq S_{P}\geq S_{S}\geq 0$$

In other words, Pauling entropy is the physical upper bound for the information capacity expressed in terms of Shannon entropy. For example, the maximum memory of ice Ih, given by its Pauling entropy expressed in bits, is 2 × 10^25^ bits per kilogram, or two terabytes per nanogram (Feistel and Ebeling [23]), see Appendix B, whether or not technical facilities may exist which permit this use.

Pauling and Shannon entropies are very similar physical quantities, they differ, however, in that Pauling entropy is given by the physical properties of a thermodynamic system while Shannon entropy is specified externally by the information-processing context, subject to technical criteria. Shannon entropy typically arises from arbitrarily declaring a certain set of physical states of the information carrier to be alternative, redundant manifestations of one and the same symbol. Shannon entropies may exist also for systems such as abstract automata, single chain molecules or laser pulses which do not necessarily possess any reasonable thermodynamic entropies.

Shannon entropy may also be termed “information entropy”, “information capacity”, “storage capacity” or “Shannon-von-Neumann entropy”.

## 6. Conclusions

Deviating from common practice in thermodynamic textbooks which consider entropy as a unique quantity that may be estimated by different methods, it is suggested here to consider the entropy formulas of Clausius, Boltzmann, Planck or Pauling as describing distinct but related properties, namely, a thermal, empirical Clausius entropy, a statistical Boltzmann entropy, and their difference, the “frozen” Pauling entropy. While similarities of those quantities are particularly striking for equilibrium states, they differ qualitatively and quantitatively for non-equilibrium states, as described by rigorous inequalities. It appears tutorially more reasonable, therefore, to introduce those entropies as different entities that coincide only under special circumstances. Pauling entropy of microstates as a kind of “potential entropy” that does not contribute to the system’s heat capacity and hence being distinct from Clausius entropy, is closely related to information capacity and forms a physical upper bound to technical Shannon entropy. Boltzmann entropy is a “total entropy” that in turn forms an upper bound to both Pauling and Clausius entropies. For equilibrium as well as for frozen non-equilibrium states, the Nernst theorem, equivalent to the 3rd law, applies to Clausius entropy of local-equilibrium systems, while equilibrium zero-point entropy, in contrast, belongs to Boltzmann and Pauling entropies. The suggested differentiation between traditional entropy concepts may be helpful for elucidating the intricate relations between entropy and information.

## Figures and Tables

**Figure A1:**
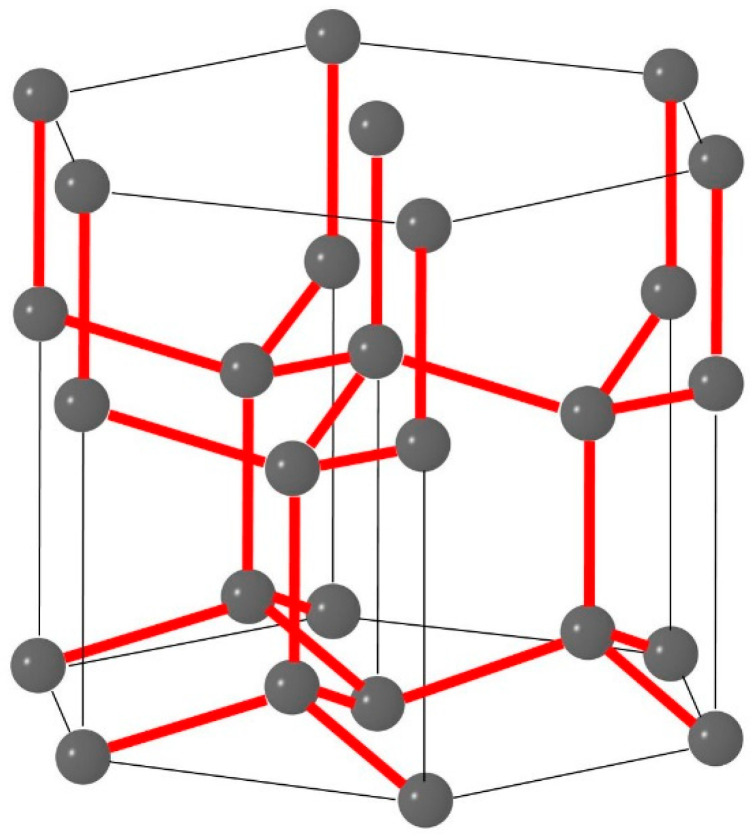
The hexagonal elementary crystal of ice Ih (Penny [88], Schulson [89], Feistel and Wagner [77]) consists of 27 oxygen (O) atoms (spheres) and 28 hydrogen (H) bonds between them (bars). Of the four H-atoms adjacent to each O-atom, two are placed closer than the other pair, thus retaining the structure of individual H_2_O molecules within the crystal lattice (Bjerrum [90]). The number of such different configurations of H-atoms possible in the crystal gives rise to the residual Pauling entropy.

**Figure A2:**
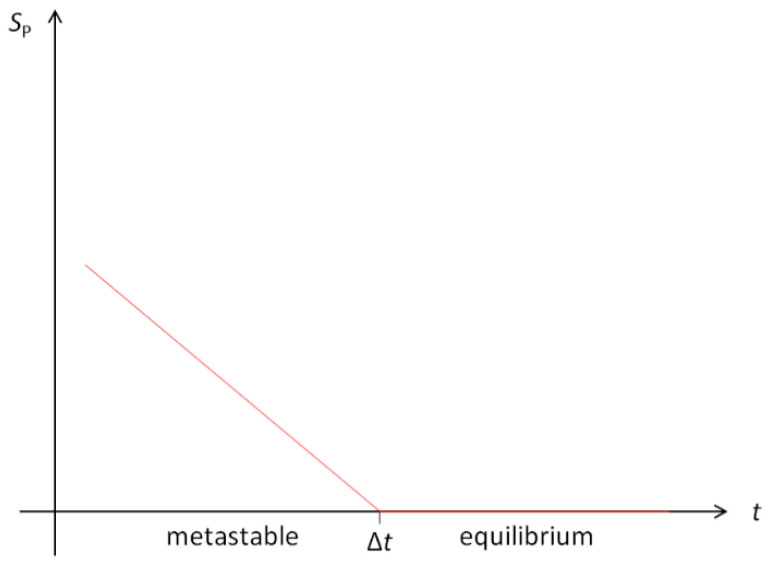
Relaxation of Pauling entropy of a droplet as a function of time, Equation (A14). At the threshold, the transition from the metastable droplet state to the 2-phase equilibrium state exhibits properties of a kinetic phase transition of the 2nd kind.

## Appendix A: On the History of the 3rd Law of Thermodynamics

Based on graphical extrapolation of observed data to *T* = 0, already in 1888 J. Clerk Maxwell ([37], p. 162) had intuitively argued that “the proper zero of entropy is that of the body when entirely deprived from heat”. More rigorously, the *Nernst Theorem* was first presented orally in 1905 at Berlin (Strehlow [68]) and originally published in 1906. It was formulated by Nernst ([10], p. 39) as “eine neue Hypothese …, nach welcher die Kurven der freien und der gesamten Energie chemischer Reaktionen zwischen lauter festen und flüssigen Körpern sich beim absoluten Nullpunkt der Temperatur tangieren”.

The paper’s argumentation starts from the Helmholtz [69] differential equation, written by Nernst ([10], p. 2) in the form:

(A1) $$A-U=T\frac{dA}{dT}$$

where *A* is “die maximale Arbeit” (likely, Helmholtz energy *F*) and *U* is “die gesamte Energie” (likely, internal energy). Generally, *F* and *A* are considered synonymous thermodynamic symbols for the “work function” or the “free energy” (Guggenheim [25], §1.32). Nernst left open the question of whether the differential in Equation (A1) is describing an isobaric or isochoric or any other process. Helmholtz ([69], p. 29) mentioned the condition “dass keine neue Wärme auf Kosten anderer Energieformen erzeugt werden dürfe”, so that isochoric rather than isobaric conditions may be assumed (Kluge and Neugebauer [70], p. 110). Additionally, Helmholtz ([69], p. 31) explained that ∂U/∂T represents the system’s heat capacity, which is correct for isochoric rather than isobaric changes. There is also a remark of Nernst ([10], p. 5) that he has in mind a container in which a chemical reaction takes place isothermally and reversibly at equilibrium while the ingredients and products of the reaction are added and removed isothermally and reversibly. This picture as well suggests an isochoric conduction of the process (A1), consistent with Equation (A2).

In more recent notation (Landau and Lifschitz [47], equation 15.5, Kluge and Neugebauer [70], equation 8.1), the Helmholtz differential equation is, similar to Helmholtz’ original formula (Helmholtz [69], p. 31, Planck [71], p. 169):

(A2) $$F-U=T{\left(\frac{\partial F}{\partial T}\right)}_{V}=-TS$$

where *S* is the entropy. The maximum work produced by a thermodynamic process is given by the change of the Helmholtz energy *F* if the process is isochoric, *V* = const, but by that of the Gibbs energy *G* = *F* + *pV* in the isobaric case, *p* = const (Landau and Lifschitz [47], equations 20.5, 20.6). As an aside, an isobaric interpretation of Equation (A1) was given by Gutzow and Schmelzer ([5], Figure 2.25), by Strehlow [68] and by Marquet [72] who identified *A* with *G* and *U* with the enthalpy *H* = *G* + *TS*.

Mathematically, Nernst ([10], p. 7) explained his fundamental hypothesis in a way that his equations will “nicht nur die Konvergenz von *dA*/*dT* und *dU*/*dT* zum Werte Null für *T* = 0 zum Ausdruck bringen, sondern gleichzeitig auch auf einen symmetrischen Verlauf von *A* und *U* in nächster Nähe des absoluten Nullpunkts hindeuten”. In Equation (A2), rigorous symmetry of *F* and *U* would imply *TS* = 0. It is worth being emphasised at this point that Nernst’s theorem refers to observations of thermal properties rather than to statistical considerations, as occasionally suggested in more recent textbooks (Pauling and Pauling [73], chapter 10). Also Fermi ([33], p. 142) interpreted Nernst’s theorem statistically when concluding that “the only circumstances under which Nernst’s theorem might be in error are those for which there exist many dynamical states of lowest energy. … Although it is not theoretically impossible to conceive of such a system, it seems extremely unlikely that such systems actually exist in nature. We may therefore assume that Nernst’s theorem is generally valid”.

In modern notation, under the assumptions made before, for T→0 the Nernst hypothesis predicts that:

(A3) $${\left(\frac{\partial F}{\partial T}\right)}_{V}=-S\to 0$$

and:

(A4) $${\left(\frac{\partial U}{\partial T}\right)}_{V}=C_{v}\to 0$$

Using quantum mechanics, Debye [74] was able to show that for crystalline solids, entropy (A3) and isochoric heat capacity (A4) follow a cubic *T*^3^ law in the vicinity of the zero point (Landau and Lifschitz [47], equations 61.7, 61.9), well consistently with Nernst’s observation of a symmetrical approach to zero of both quantities (Kluge and Neugebauer [70]). There, the difference between isochoric and isobaric heat capacities disappears even faster with falling temperature:

(A5) $$C_{p}-C_{v}\;\sim \;T^{7}$$

The physical reason for the cubic law is that vibrational modes (phonons) have statistical properties very similar to Planck’s thermal radiation (Landau and Lifschitz [47], equation 61.6). “Bei Temperaturen, die niedrig genug sind, wird der Inhalt eines Festkörpers an Schwingungsenergie proportional zu *T*^4^ sein, ganz analog zum Stefan-Boltzmannschen Strahlungsgesetz” (Debye, quotation from Kittel [1], p. 295). Because the energy of both is proportional to *T*^4^, entropy and heat capacity, Equations (A3) and (A4), are both proportional to *T*^3^, a law that is fairly independent of the body’s microscopic structural details, except for metals (Kittel [1], equation 14.37). At low temperatures, typically about 10 K, thermal energy falls below the finite excitation energy of other quantum states, and the cubic law starts dominating the heat capacity. For example, the reference equation of state for hexagonal ice (Feistel and Wagner [75,76,77,78], Feistel [18]) obeys those Debye [74] and Grüneisen [79] laws, consistently with experimental data. For amorphous solids, the transition temperature may be even lower (Gutzow and Schmelzer [5], p. 209).

Note that despite Equation (A3), Nernst [10] *did not* draw any explicit conclusion regarding the value of entropy at the zero point. This task was eventually taken over by Planck ([9], p. 268), as intentionally quoted here literally at greater length: “Ein Theorem, welches W. Nernst im Jahre 1906 aufgestellt hat …, läßt sich dahin formulieren, daß beim Nullpunkt der absoluten Temperatur die Entropie eines jeden chemisch homogenen festen oder flüssigen Körpers einen bestimmten, von dem Aggregatzustand und von der speziellen chemischen Modifikation unabhängigen Wert besitzt. [Diese Fassung des Theorems ist inhaltlich etwas weitergehend als die von Nernst a.a.O. selber gegebene, nach welcher für *T* = 0 die Differenz der Entropien eines solchen Körpers in zwei Modifikationen gleich Null ist.] … Zunächst ist leicht einzusehen, daß man, da der Wert der Entropie eine willkürliche additive Konstante enthält, jenen für *T* = 0 eintretenden Wert unbeschadet der Allgemeinheit gleich Null setzen kann, so daß das Nernstsche Wärmetheorem nun lautet:

*Beim Nullpunkt der absoluten Temperatur besitzt die Entropie eines jeden chemisch homogenen festen oder flüssigen Körpers den Wert Null.*

Damit ist über die additive Konstante *a* der Entropie aller chemisch homogenen Substanzen in allen Zuständen eindeutig verfügt, insofern jede Substanz im festen oder flüssigen Aggregatzustand bei der Temperatur Null existenzfähig (wenn auch nicht stabil) ist, und man kann von nun an in diesem Sinne von einem absoluten Wert der Entropie sprechen.“

[English translation: “A theorem that was stated by W. Nernst in the year 1906 … may be formulated in a way that at the zero point of the absolute temperature, the entropy of any chemically homogeneous, solid or liquid body possesses a certain value that is independent of the phase and the particular chemical modification. (From its contents, this formulation of the theorem goes slightly beyond that given elsewhere by Nernst himself, after which at *T* = 0 the difference between the entropies of such a body in two modifications is zero.) … Because the entropy value includes an arbitrary additive constant, it is easily recognized that without loss of generality, the value at *T* = 0 may be set to zero, so that Nernst’s heat theorem then reads:

*At the zero point of the absolute temperature, the entropy of any chemically homogeneous solid or liquid body possesses the value of zero.*

This way, the additive constant *a* of the entropy of all chemically homogeneous substances in any states is uniquely determined, as far as that substance is able to exist (even if not stably) in solid or liquid phase at zero temperature, and in this sense one may then speak about an absolute value of the entropy.”]

It should be mentioned here that in one point the modern perspective deviates from Planck’s visionary statement. Most specialists agree that both solid H_2_O phases ice Ih and ice XI can exist at *T* = 0 under ambient pressure, but they possess different zero-point entropies (see Appendix B). If this counterexample is correct, the invariance of zero-point entropy with respect to changes of density or pressure is not valid across phase transitions (Feistel and Ebeling [23], p. 376), in contrast to what Planck had assumed originally by saying that at the zero point the entropy of any chemically homogeneous, solid or liquid body possesses a certain value that is *independent* of the phase.

Note, however, that the actual value of the absolute entropy is irrelevant for the description of natural processes, as emphasized already by Planck [9,71], because only differences of energies, entropies etc. appear in the related equations. For this reason, international standard equations for thermodynamic properties of water, seawater, ice or humid air are expressed in entropies that formally vanish at experimentally convenient reference points at which the measurement uncertainty is sufficiently small, such as the triple point of water, rather than at *T* = 0 (Feistel [18]). The convenient technical setting of the value for the zero-point entropy is consistent with all available reliable thermodynamic measurements and is in no way related to the fact that “the general theoretical proof of the fact that the state of a system with the smallest energy is at the same time one of zero entropy has not yet been given” (Simon [39]). In Appendix B, the reasons will be considered more closely.

For clarity it should be emphasized finally again that in this paper, the Nernst theorem is identified with the 3rd law, both valid, without any known exceptions among local-equilibrium systems, for empirical thermodynamics and thermal processes, and for the Clausius entropy. On the contrary, residual or zero-point entropy are considered here as quantities that belong to statistical thermodynamics, or Boltzmann and Pauling entropies, and do not contribute to measurable heat capacities. In the literature, however, the 3rd law, the Nernst theorem and the residual entropy are often set in mutual relations other than here. For example, Kittel [1], p. 144) writes that “Der dritte Hauptsatz … stellt eine Annahme über die Invarianz der Entartung des Grundzustandsniveaus … dar“, thus associating the 3rd law with statistical thermodynamics, in contrast to Nernst’s original hypothesis. Similarly, the “third principle” as formulated by Guggenheim ([25], p. 47) may be mentioned whose applicability is restricted to statistical thermodynamics only. Empirically measurable violations of Nernst’s theorem such as those described by Gutzow and Schmelzer ([5], Figure 2.25) are not related to the degeneracy of the quantum ground state but rather to non-equilibrium microstate populations trapped in excited quantum states. Such systems are not considered in this paper.

When discussing the validity of the “Nernst theorem”, or synonymously, of the “3rd law” for certain systems, it is important to distinguish two different formulations which coincide at equilibrium but may fall apart otherwise. The original Nernst theorem, see above, is about the symmetry of two energy curves, *A* and *U*, Equation (A1), in the vicinity of *T* = 0. Their shapes can be investigated experimentally in the framework of empirical thermodynamics, and breaking this symmetry by some systems such as glasses had been measured extensively (Gutzow and Schmelzer [5], Figure 2.25). On the other hand, Planck’s formulation of the Nernst theorem, or the 3rd law, saying that entropy vanishes at *T* = 0, can be verified for local-equilibrium metastable states only statistically by investigating the quantum ground-state degeneracy.

## Appendix B: On the History of the Zero-Point Entropy

While the Clausius entropy can relatively easily be computed from measured heat capacities of available substances, estimating the Boltzmann entropy requires complicated mathematics of statistical thermodynamics, a task which in general poses a serious challenge. For the special case of ideal gases, Boltzmann [43] and Planck [8] were very successful in statistically deriving thermodynamic equations of state that fit the experimental data. At the time Einstein [4] suspected the possibility of inconsistencies between the empirical and statistical approaches, there was no reliable experimental evidence for definitely deciding this question. Later it became possible, however, to count quantum states from spectroscopic data in the infrared range and to compare this way the independently derived experimental values of *S*_C_ and *S*_B_. At 298.1 K and normal pressure, Giauque and Ashley [80] found the molar Boltzmann entropy of water vapour to be 47.92 cal deg^−1^ mol^−1^, corresponding to a specific Boltzmann entropy of $s_{B}^{V}=11\;131\;{J\;\mathrm{kg}}^{-1}{\;K}^{-1}$. This value was later recalculated more precisely by Gordon [81] to be 45.101 cal deg^−1^ mol^−1^, or $s_{B}^{V}=10\;476\;{J\;\mathrm{kg}}^{-1}{\;K}^{-1}$. A related modern CODATA value for the specific Boltzmann entropy of liquid water at 298.15 K and 0.1 MPa is 69.95(3) J mol^−1^ K^−1^, or $s_{B}^{L}=3883\left(2\right)\;{J\;\mathrm{kg}}^{-1}{\;K}^{-1}$, as reported by Cox et al. [82].

On the other hand, computed from the 3rd law and the latent heat and the temperature integral over the heat capacity of ice as well as the ideal-gas Sackur-Tetrode equation for water vapour, Giauque and Stout [83] obtained for the molar Clausius entropy of water vapour a value of 44.28 cal deg^−1^ mol^−1^, or $s_{C}^{V}=10\;285\;{J\;\mathrm{kg}}^{-1}{\;K}^{-1}$ (for a quantitative example of such a calculation, see Guggenheim ([25], §4.04), or Marquet ([72], equation 3). Being about 2 % lower than that of Gordon [81], this result represents a significant difference exceeding the experimental uncertainty. This discovery was finally awarded with the Nobel Prize in Chemistry 1949 to William F. Giauque. The unexpectedly inconsistent results found experimentally for the Boltzmann and Clausius entropies of water were subsequently explained by Pauling [73], allegedly quickly calculated by him on a beer mat, taking into account the degeneracy of the ground state of the molecular structure of ice Ih (Fletcher [84]), see Figure A1. Pauling’s first estimate was mathematically improved by Nagle [85] to result in a theoretical specific zero-point entropy of $s_{P}^{0,\mathrm{Ih}}=189.13\pm 0.05\;{J\;\mathrm{kg}}^{-1}{\;K}^{-1}$, a value that became known as the Pauling residual entropy of ice Ih. In excellent agreement with the statistical result, the latest, improved experimental value for the zero-point entropy is $s_{P}^{0,\mathrm{Ih}}=189.3\pm 10.6\;{J\;\mathrm{kg}}^{-1}{\;K}^{-1}$ (Haida et al. [86], Petrenko and Whitworth [87]).

Implementing the Pauling-Nagle residual entropy in the international standard equations of state for ice Ih and fluid water (Wagner and Pruß [91], Feistel and Wagner [78], Feistel [18]) results in a value for the specific Boltzmann entropy of liquid water at 298.15 K and 0.1 MPa of $s_{C}^{L}+s_{P}^{0,\mathrm{Ih}}=3883.7\;{J\;\mathrm{kg}}^{-1}{\;K}^{-1}$, being perfectly consistent with the experimental CODATA figure of $s_{B}^{L}=3883\left(2\right)\;{J\;\mathrm{kg}}^{-1}{\;K}^{-1}$ (Cox et al. [82]).

There exists the hypothesis that ice Ih is metastable at temperatures below about 100 K (Johari and Jones [92]). The stable phase of ice at the zero point is assumed to be ice XI, a proton-ordered crystal without residual entropy (Singer et al. [93]). Note that such an assumed phase transition is inconsistent with formulations of the 3rd law that claim the zero-point entropy to be not only invariant with respect to changes of density or pressure, but also against phase transitions, modifications or different states of aggregation, including metastable condensed phases if those states may exist at least (Planck [9], Gutzow and Schmelzer [5,7]), see Appendix A. Again, by contrasting the distinct entropies, the invariance of the zero-point entropy with respect to alternative molecular configurations is assumed to be obeyed by the Clausius entropy but may be violated by the Boltzmann entropy. Various zero-point entropies for substances other than water have meanwhile be determined (Gutzow and Schmelzer [5,7]).

## Appendix C

A theoretical method for estimating the Pauling entropy, Equation (21), is by constructing an arbitrary fictitious future process, possibly involving suitable catalysts, that leads from the given frozen non-equilibrium state to the associated final equilibrium state, consistent with the boundary conditions, and then summing up the entropy production along the path. As entropy is a state function of local-equilibrium systems such as the initial and the final state, the value of the resulting integral will not depend on the specifics of the transition process.

Clouds constitute a key element of the terrestrial climate system (Lamb and Verlinde [94], Pelkowski and Frisius [95], Randall [96], Feistel et al. [97]). Suspended water droplets in the atmosphere represent a metastable non-equilibrium state that slowly relaxes toward equilibrium by Ostwald ripening (Ostwald [98], Schmelzer [99,100], Mahnke and Feistel [101], Schmelzer and Ulbricht [102]). In this irreversible process, the liquid of smaller droplets evaporates faster than it does condense out of the vapour, but vice versa for larger droplets, so that an overall coarse graining takes place among the droplets, accompanied by a gradual reduction of the total droplet surface area.

Here we study a simplified model of that process with a single droplet immersed in saturated vapour, the latter being in equilibrium with a liquid bulk phase across a planar interface, like fog over a lake. The droplet mass is much smaller than the model’s remaining total mass. During the slow relaxation process, the system is assumed to remain thermally homogeneous and the liquid to be incompressible. The evaporation from the droplet is compensated by an equal rate of precipitation to the buffer liquid. Assumingly, the total amounts of liquid and vapour remain the same throughout the ripening.

The chemical potential of a liquid in a droplet with the radius *r* is approximately (Thomson [103], Gutzow and Schmelzer [5], Lamb and Verlinde [94]):

(A6) $$\mu_{L}^{\left(r\right)}\left(r,T,p\right)=g^{L}\left(T,p\right)+\frac{2\sigma v}{r}$$

where *σ* is the surface tension, *v* is the specific volume and *g*^L^ the specific Gibbs energy of the liquid, for example that of water (Wagner and Pruß [91], Feistel [18]).

The Onsager force driving evaporation from the droplet to the surrounding vapour can be assumed to be proportional to the chemical potential difference between droplet, $\mu_{L}^{\left(r\right)}$, and vapour, *µ*_V_ (DeGroot and Mazur [17], Feistel and Ebeling [23], equations 3.87, 3.97):

(A7) $$X=\frac{\mu_{L}^{\left(r\right)}}{T}-\frac{\mu_{V}}{T}=\frac{1}{T}\frac{2\sigma v}{r}$$

Here, the chemical potential *µ*_V_ equals the specific Gibbs energy of the saturated vapour, *g*^V^:

(A8) $$\mu_{V}=g^{V}=g^{L}$$

The related evaporation mass-flux density, *j*^(*r*)^, is:

(A9) $$j^{\left(r\right)}=\kappa X=\frac{\kappa }{T}\frac{2\sigma v}{r}$$

For instance, the Onsager coefficient κ for the evaporation of water can be estimated to a climatological mean value of $\kappa \approx 0.4\cdot 10^{-6}{\;\mathrm{kg}}^{2}{\;K\;J}^{-1}{\;m}^{-2}{\;s}^{-1}$ in the terrestrial hydrological cycle (Feistel and Ebeling [23]).

Entropy production, *P*, is a bilinear expression in terms of Onsager forces and fluxes (DeGroot and Mazur [17], Pelkowski [104], Gassmann and Herzog [105]), see Equation (9). Integrated over the droplet surface, *A*, the entropy production of the mass flow across the difference of chemical potentials is:

(A10) $$P=j^{\left(r\right)}AX=\kappa AX^{2}=\kappa \;4\pi r^{2}{\left(\frac{1}{T}\frac{2\sigma v}{r}\right)}^{2}=16\pi \kappa {\left(\frac{\sigma v}{T}\right)}^{2}$$

This entropy production appears to be independent of the droplet’s size and age.

For the computation of the Pauling entropy, Equation (21), we omit for simplicity any possible zero-point entropy, $S_{P}^{0}=0$. Because *P* is time-independent, the droplet’s lifetime Δ*t* is expected to be finite:

(A11) $$S_{P}\left(t\right)=\int_{t}^{\infty }Pdt=\left(\Delta t-t\right)P$$

To find Δ*t*, the mass balance of the droplet is considered. The mass of the droplet is $m=\frac{4}{3}\pi r^{3}/v$, and the related mass loss by evaporation is:

(A12) $$\frac{dm}{dt}=\frac{4\pi }{v}r^{2}\frac{dr}{dt}=-j^{\left(r\right)}A=-\frac{\kappa }{T}\frac{2\sigma v}{r}4\pi r^{2}$$

This differential equation for *r*(*t*) is easily integrated to provide the decay law of the radius:

(A13) $$r\left(t\right)=\sqrt{r_{0}^{2}-\frac{4\kappa v^{2}\sigma }{T}t}=r_{0}\sqrt{1-\frac{t}{\Delta t}}$$

or similarly, for the shrinking surface area:

(A14) $$A\left(t\right)=A_{0}\left(1-\frac{t}{\Delta t}\right)$$

where *r*_0_, *A*_0_, respectively, are the initial radius and surface. The droplet disappears after the finite lifetime of:

(A15) $$\Delta t=\frac{r_{0}^{2}T}{4\kappa v^{2}\sigma }$$

Equation (A13) is known as the *R*^2^ *law* (or *D*^2^ *law*) of Maxwell and Langmuir (Sobac and Brutin [106], p. 106). “The apparent linearity of *r*^2^(*t*) is retained even for relatively small droplets and relatively fast evaporation/condensation, since the *r*^2^(*t*) form is not very sensitive to kinetic effects” (Jakubczyk et al. [107], p. 715).

Finally, from Equations (A10)–(A15), the Pauling entropy of the droplet results to be proportional to its instantaneous surface area:

(A16) $$S_{P}\left(t\right)=P\Delta t\left(1-\frac{t}{\Delta t}\right)=\frac{\sigma A_{0}}{T}\left(1-\frac{t}{\Delta t}\right)=\frac{\sigma }{T}A\left(t\right).$$

It is remarkable, see Figure A2, that at the critical point, t=Δt, the model approaches equilibrium abruptly rather than asymptotically by exponential relaxation. The threshold has properties of a kinetic phase transition of the 2nd kind; the transition occurs in a continuous manner but is accompanied by the symmetry-breaking disappearance of the droplet.

It follows from Equation (A16) that the entropy production, Equation (A10), may be written in compact form:

(A17) $$TP=-\sigma \frac{dA}{dt}=\frac{\sigma A_{0}}{\Delta t}\;\mathrm{if}\;t<\Delta t,\;TP=0\;\mathrm{otherwise}$$

The result (A16) is consistent with the entropy formula of Schmelzer and Ulbricht ([108], equation 2.5) obtained by different methods, in which the term σA/T proves to be the dominant contribution (Schmelzer [109]). Estimating the Pauling entropy of a non-equilibrium spatial configuration by the surface area may, in a first approximation, also be a suitable measure for other frozen structures such as coins (Figure 1). It is expected that the droplet entropy formula is independent of the relaxation route to equilibrium, in particular, of special evaporation rate laws. The Pauling entropy σA/T depends only on surface properties but neither on those of the droplet interior nor on rate coefficients of the relaxation process. In this respect, interestingly, the Pauling entropy of a droplet has some similarity to the Bekenstein [110] entropy of a black hole.
